# Comparison of Efficacy of Acupuncture-Related Therapy in the Treatment of Postherpetic Neuralgia: A Network Meta-Analysis of Randomized Controlled Trials

**DOI:** 10.1155/2022/3975389

**Published:** 2022-10-14

**Authors:** Haiyan Wang, Renhong Wan, Shuai Chen, Haiyan Qin, Wei Cao, Luqiang Sun, Yunzhou Shi, Qianhua Zheng, Ying Li

**Affiliations:** ^1^School of Acupuncture-Moxibustion and Tuina, Chengdu University of Traditional Chinese Medicine, Chengdu 610075, China; ^2^Tianjin University of Traditional Chinese Medicine, Tianjin 301617, China

## Abstract

**Background:**

Postherpetic neuralgia (PHN) is the most common sequela of herpes zoster, and the efficacy of the treatment regimens recommended in the guidelines is not entirely reliable. Acupuncture and moxibustion are widely used complementary alternative therapies that have a positive effect on the treatment of PHN. However, there are various forms of acupuncture and moxibustion, and there are differences in efficacy between the different forms.

**Methods:**

The retrieval work of randomised controlled trials (RCTs) of acupuncture for PHN in English databases (including PubMed, Cochrane Library, Embase, Web of Science) and Chinese databases (including China National Knowledge Infrastructure (CNKI), WeiPu database, WanFang database, and China Biomedical Literature Database) were conducted from the time of database creation to June 2022. Literature screening, data extraction, and evaluation of risk of bias for the included studies were carried out independently by two researchers, and data analysis was performed using Stata 14.2 software.

**Results:**

A total of 30 RCTs including 2138 patients with PHN were included. In terms of pain improvement, acupoint embedding + Western medicine group, bloodletting-cupping group, and bloodletting-cupping + Western medicine group ranked top. In terms of total efficiency, acupuncture + Western medicine group, bloodletting-cupping + Western medicine group, and acupoint embedding group ranked top. There were no statistically significant differences in the incidence of adverse events between treatment regimens.

**Conclusions:**

In a comprehensive comparison of the outcome indicators of 14 different treatment regimens, we considered acupoint injection + Western medicine and bloodletting-cupping + Western medicine to be the best combinations for the treatment of PHN. Due to the limitations of the study, the above conclusions still need to be validated in further multi-centre, large-sample prospective randomised controlled clinical trials.

## 1. Introduction

PHN is a neuropathic pain that persists for 1 month or more after the healing of the herpes zoster (HZ) rash and is the most common complication of herpes zoster [1]. The nature of pain in PHN is varied and can be burning, electric shock, knife-like or hand-needle pain, intermittent or continuous [2]. Approximately 18∼41% of patients with HZ experience PHZ, a painful condition that can last for months or even years [3], during which physical, emotional, and social functioning are affected [4]. PHZ also adds to the medical burden at the individual and societal level [1].

Current treatment for PHN includes both pharmacological and interventional treatments, with pharmacological treatment being the most basic and commonly used method [5]. The drugs currently recommended for first-line treatment of PHN are calcium channel modulators (e. g. pregabalin, gabapentin, tricyclic antidepressants (TCAs) and lidocaine patch 5%) [6, 7]. However, these drugs are not suitable for long-term use and their efficacy is not entirely reliable [8]. Other treatments include opioid analgesics, tramadol, topical capsaicin and botulinum toxin type A, but the long-term efficacy and safety of these drugs is uncertain [1, 9]. Therefore, how to optimise PHN treatment strategies is a matter of concern to clinicians.

Acupuncture-related therapy, based on meridian theory, is a traditional treatment modality of Chinese origin that plays an important role in the field of complementary and alternative therapies. A 2010 survey of global research trends in acupuncture [10] showed that pain management with acupuncture has been the most prevalent area of research and that acupuncture-related therapies have been widely used in the treatment of joint, muscle, and nerve-related pain and are safe and effective [11–14]. A large number of clinical studies on acupuncture-related therapies for the treatment of PHN have been conducted in China and abroad, and there are various forms of acupuncture, including acupuncture, moxibustion, electroacupuncture, fire acupuncture, and blood-letting [8]. Recent systematic evaluations have confirmed that acupuncture-related therapies (including fire acupuncture, acupuncture, electroacupuncture, and moxibustion) are effective for PHN and that they can reduce pain intensity, relieve anxiety, and improve quality of life in patients with PHN [15–17]. As these studies have mainly focused on the comparison of acupuncture therapies with traditional drug therapies, there is a lack of comparison of efficacy between different acupuncture therapies, and therefore, there is still controversy in clinical practice as to which acupuncture therapy is the best choice. Network meta-analysis (NMA) is a further development of traditional pairwise meta-analysis [18]. Based on current clinical research data, the NMA can complete both direct and indirect comparisons between different acupuncture therapies and further synthesise the results of direct and indirect comparisons to produce a ranking of the efficacy of different acupuncture treatments [11]. This study therefore used Network Meta-analysis to compare the efficacy of different acupuncture and moxibustion therapies in the treatment of PHN and to provide evidence for choosing the optimal combination for the clinical treatment of PHN.

## 2. Methods

### 2.1. Study Registration

This network meta-analysis was conducted according to the Preferred Reporting Items for Systematic Reviews and Meta-Analyses for NMA guidelines [19]. This research programme has been registered on PROSPERO at https://www.crd.York.ac.uk/prospero/#recordDetails; registration number: CRD42022324870.

### 2.2. Inclusion and Exclusion Criteria

#### 2.2.1. Type of Study

This study included parallel randomised controlled trials (RCTs) published in Chinese or English, which were subject to the limitations of the intervention. There was no requirement for the study to be blinded.

#### 2.2.2. Study Population

Patients included in the study met the American Academy of Family Physicians' diagnostic criteria for PHN [2], or other accepted diagnostic guidelines for pain lasting 30 days to more than 6 months after healing of the HZ lesions. There were no restrictions on the gender or age of the patients.

#### 2.2.3. Interventions

The treatment group was treated with different acupuncture therapies alone or in combination with Western medicine, which were defined as acupoint stimulation techniques guided by the meridian theory of Chinese medicine, including any of the following therapies: conventional acupuncture, warm acupuncture, electroacupuncture, fire acupuncture, bloodletting-cupping, moxibustion, acupoint burial, acupoint injection; the control group was Western medicine, which had to be first-line drugs recommended by guidelines for the treatment of PHN [6], including calcium ion channel modulators (e. g. pregabalin, gabapentin, tricyclic antidepressants (TCAs) and lidocaine patch 5%).

#### 2.2.4. Outcome Indicators

Primary outcome indicators: the primary objective of this study was to assess pain control, and pain measures included the Visual Analogue Scale (VAS) and Numerical Rating Scale.

*Secondary outcome indicators*. ① The efficiency rate, which was based on the evaluation of the overall efficacy and the evaluation criteria based on the Criteria of Diagnosis and Therapeutic Effects of Diseases and Syndromes in Traditional Chinese Medicine [20] and the Guiding Principles for Clinical Research of New Chinese Medicines [21]; ② the occurrence of adverse effects.

*Exclusion criteria*. ① The presence of other painful conditions in the study population; ② lack of primary outcome indicators; ③ treatment protocols that included a combination of two or more acupuncture therapies, such as acupuncture combined with moxibustion, electroacupuncture combined with moxibustion, etc.; ④ duplicate published studies; and ⑤ studies for which complete data were not available in the article and relevant data were still not available after contacting the authors.

### 2.3. Literature Search Strategy

We searched Chinese databases (China National Knowledge Infrastructure (CNKI), WanFang Data, VIP, and CBM) and English databases (PubMed, Embase, Web of Science, and the Cochrane Library) for RCTs of acupuncture-related therapies for PNH. We used the method of subject terms combined with free words, and the Chinese search terms included “Shou Zhen” (acupuncture), “Dian Zhen” (electroacupuncture), “Wen Zhen Jiu” (warm acupuncture), “Huo Zhen” (fire acupuncture), “Ci Luo Ba Guan” (bloodletting-cupping), “Ai Jiu” (moxibustion), “Xue Wei Mai Xian” (acupoint embedding), “Xue Wei Zhu She” (acupoint injection), and “Dai Zhuang Pao Zhen Hou Yi Shen Jing Tong” (post-herpetic neuralgia). English search terms include “acupuncture,” “electroacupuncture,” “warm needle,” “fire needle,” “blood-letting,” “moxibustion,” “acupoint embedding,” “acupoint injection,” “Postherpetic neuralgia,” “PHN.” The PubMed database search strategy is shown in Table 1.

### 2.4. Literature Selection

The retrieved literature was imported into EndNote X9 software and checked for duplicates. After excluding duplicates, an initial screening was carried out by reading the abstracts to exclude irrelevant studies, and the remaining studies were further assessed by reading the full text, and finally data extraction was completed for those studies that met the inclusion criteria. All of the work above was done by two independent researchers, and where there was disagreement between the two researchers, a third researcher assisted in the judgement. Data extraction included title, author, date of publication, sample size, details of the intervention, details of the control measure, duration of treatment, and outcome indicators. For multiarm studies reporting different types of acupuncture interventions, data were extracted from all relevant arms. For pain scores, the mean and standard deviation of baseline and posttreatment change scores (defined as the baseline score minus the posttreatment score) were taken.

### 2.5. Risk of Bias Evaluation of Included Studies

Two independent researchers evaluated the included studies back-to-back by means of the Cochrane Systematic Evaluation Manual version 5.1.0 RCT Risk of Bias Assessment Tool [22]. The elements of the evaluation included random sequence generation, outcome allocation concealment, blinding of participants and personnel, blinding of assessment, incomplete outcome data, selective reporting bias and other bias. The final decision was “high risk,” “low risk,” or “unclear”.

### 2.6. Statistical Analysis

#### 2.6.1. Direct Comparison

Direct comparative meta-analysis was performed using Stata 14.2 software. For continuous variables, analysis was carried out using the standard mean difference (SMD), and for dichotomous variables, relative risk (RR) was used. Heterogeneity between the results of the included studies was analysed using the *χ*^2^ test, while the magnitude of heterogeneity was determined quantitatively in conjunction with *I*^2^. If *P* ≥ 0.10, *I*^2^ < 50%, there was no significant heterogeneity between studies and a fixed-effects model was used for meta-analysis; if *P* < 0.10, *I*^2^ ≥ 50%, heterogeneity between studies was considered significant and a random-effects model was used for meta-analysis.

#### 2.6.2. Reticulated Meta-Analysis

The Stata 14.2 software was used to plot the net relationship between direct and indirect comparisons between the outcomes of the different treatment measures. For studies with three or more arms, they were first divided into pairwise contrast combinations. The included studies were also tested for consistency and non-consistency, and local inconsistency was tested using the nodal split method; *P* > 0.05 was considered good for consistency. For each outcome indicator, the efficacy of the interventions was ranked using the surface under the cumulative ranking (SUCRA) values. The publication bias of included studies was tested by comparison-corrected funnel plots.

## 3. Results

### 3.1. Literature Search Results

**Figure 1:**
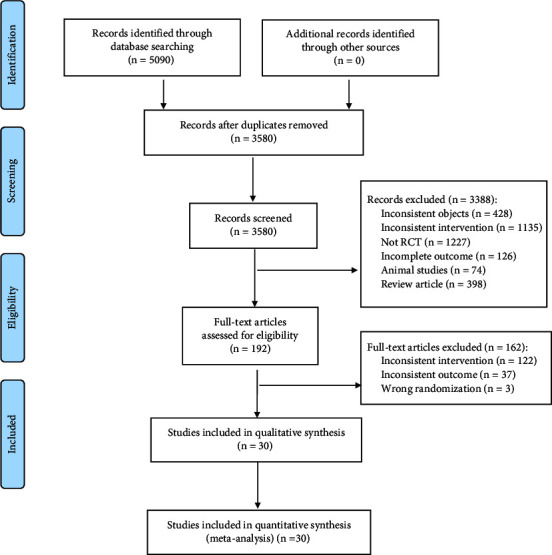
Literature screening process.

A total of 5090 relevant literature was retrieved, and after initial screening and re-screening, 30 RCTs were finally included, comprising a total of 2138 patients. The literature screening process is shown in Figure 1.

### 3.2. Basic Characteristics of the Included Studies

Of the 30 studies included, three involved electroacupuncture; one involved electroacupuncture combined with Western medicine; two involved warm acupuncture, six involved bloodletting-cupping; four involved bloodletting-cupping combined with Western medicine; four involved acupuncture; four involved acupuncture combined with Western medicine; three involved fire acupuncture; one involved acupoint injection, two involved acupoint injections combined with Western medicine; two involved acupuncture burials; one involved acupuncture burial combined with Western medicine; one involved blood prick; and 30 involved Western medicine. There were two three-armed studies [23, 24], one four-armed study [25], and 27 two-armed studies [26–52]. All studies reported pain scores [23–52], 23 studies reported overall effectiveness [23, 25, 26, 28–38, 40, 42–44, 46, 47, 49, 50, 52] and 14 studies [25, 26, 31, 33, 36, 39, 43–45, 47–49, 51, 52] reported adverse effects. The basic characteristics of the included studies are shown in Table 2 and the characteristics of the interventions are shown in Table 3.

### 3.3. Results of the Risk of Bias Evaluation of the Included Studies

**Figure 2:**
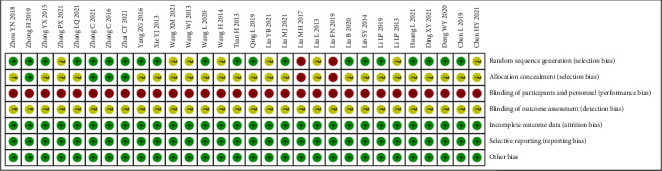
Results of the risk of bias evaluation.

① Random sequence generation: 17 studies [23, 25, 28, 29, 31, 32, 35, 36, 39–41, 44, 45, 48, 49, 51, 52] used random number tables, three studies [30, 34, 46] used computer-generated random numbers, two studies [37, 42] used consultation sequences and the remaining eight studies [24, 26, 27, 33, 38, 43, 47, 50] referred to “random” only. ② Allocation concealment: 4 studies [23, 30, 48, 52] used sealed opaque envelopes; 2 studies [37, 42] used order of attendance and the remaining 24 studies did not mention allocation concealment. ③ Blinding of patients, trialists: none of the studies were double-blinded due to the limitations of the intervention modality. ④Blinding of outcome assessors: none of the studies mentioned blinding of outcome assessors; ⑤Incomplete outcome data, selective reporting, other bias: all studies had complete outcome data, with no selective reporting or other bias. The results of the risk of bias evaluation are shown in Figure 2.

### 3.4. Directly Compared meta-Analysis Results

#### 3.4.1. Pain Scores

In a direct comparison regarding pain scores, meta-analysis showed that the pain score of warm acupuncture, electroacupuncture, electroacupuncture + Western medicine, acupuncture + Western medicine, bloodletting-cupping, bloodletting-cupping + Western medicine, fire acupuncture, and acupoint embedding groups were superior to that of the Western medicine group (*P* < 0.05), while the scores in acupuncture and acupuncture point injection + Western medicine groups did not differ from the Western medicine group (*P* > 0.05). Descriptive analysis showed that both the acupoint injection and acupoint embedding groups were superior to the Western medicine group (*P* < 0.05). The acupuncture group was superior to the electroacupuncture group, the acupuncture + Western medicine group was superior to the acupuncture group (*P* < 0.05), and there was no difference between the fire acupuncture + Western medicine group and the Western medicine group, the fire acupuncture group and the bloodletting-cupping group compared to the acupuncture group (*P* > 0.05), see Table S2 in the supplementary material.

#### 3.4.2. Total Efficiency

In a direct comparison regarding pain scores, meta-analysis showed that the acupuncture, acupuncture + Western medicine, fire acupuncture, bloodletting-cupping, acupoint embedding groups were all superior to the Western medicine group (*P* < 0.05), with no difference in the electroacupuncture group compared to the Western medicine group. Descriptive analyses of acupoint embedding + Western medicine compared with Western medicine, fire acupuncture, bloodletting-cupping, and electroacupuncture compared with acupuncture showed no difference (*P* > 0.05), see Table S2 in the supplementary material.

#### 3.4.3. Adverse Reactions

In a direct comparison regarding adverse reaction rates involving 10 interventions (warm acupuncture, acupuncture, acupuncture + Western medicine, bloodletting-cupping, bloodletting-cupping + Western medicine, electroacupuncture, electroacupuncture + Western medicine, acupoint injection, acupoint injection + Western medicine, and acupoint embedding) versus Western medicine, none of their adverse reaction rates differed from those of Western medicine (*P* > 0.05), see Table S2 in the supplementary material.

#### 3.4.4. Heterogeneity Analysis

In the meta-analysis of direct comparisons, there was heterogeneity in some of the results. Analysis of the raw data revealed possible methodological heterogeneity due to the inclusion of studies with less description of blinding and allocation concealment, as well as possible clinical heterogeneity due to factors such as inclusion of populations, acupuncture points, and manipulation methods, but as the original studies did not specify these details and the small number of studies for some of the outcomes, further subgroup analysis could not be performed to explore sources of heterogeneity. However, we found by sensitivity analysis that the results were stable after excluding either study; see sensitivity analysis figures in the supplementary material (Figure S1∼S19). We could therefore ignore this heterogeneity and used a random-effects model for Meta-analysis.

### 3.5. Comparative Results of Reticulated Meta-Analysis

#### 3.5.1. Evidence Network Map

**Figure 3:**
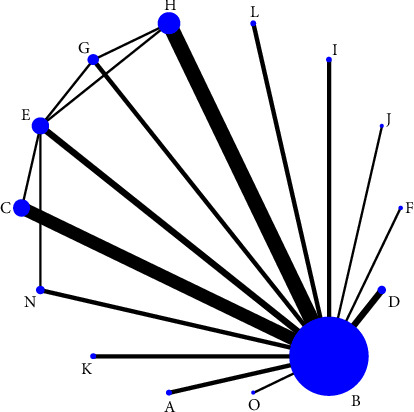
Evidence network graph for pain scores in a reticulated meta-analysis of different acupuncture therapies for PHN. *Notes*. (a) Warm acupuncture; (b) western medicine; (c) acupuncture + western medicine; (d) bloodletting-cupping + western medicine; (e) acupuncture; (f) fire acupuncture + western medicine; (g) fire acupuncture; (h) bloodletting-cupping; (i) acupoint injection + western medicine; (j) acupoint injection; (k) electroacupuncture + western medicine; (l) acupoint embedding; (n) electroacupuncture; and (o) acupoint embedding + western medicine.

**Figure 4:**
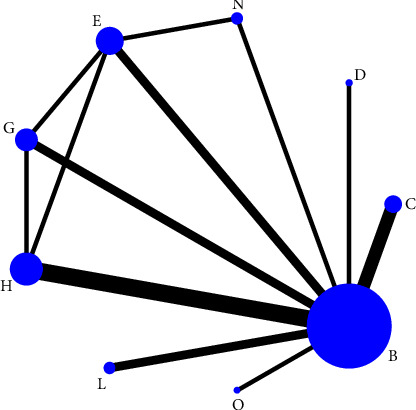
Evidence network diagram of the total efficiency of different acupuncture therapies for PHN in reticulated meta-analysis. *Notes*. (b) western medicine; (c) acupuncture + western medicine; (d) bloodletting-cupping + western medicine; (e) acupuncture; (g) fire acupuncture; (h) bloodletting-cupping; (l) acupoint embedding; (n) electroacupuncture; and (o) acupoint embedding + western medicine.

**Figure 5:**
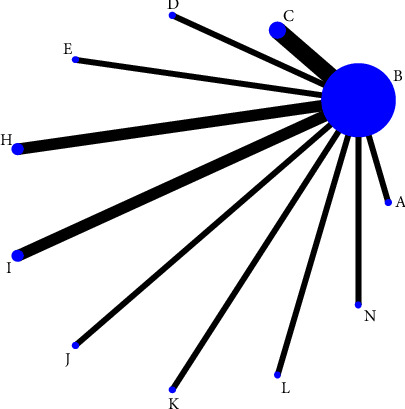
Evidence network diagram for adverse effects of reticulated meta-analysis of different acupuncture treatments for PHN. *Notes*. (a) warm acupuncture; (b) western medicine; (c) acupuncture + western medicine; (d) bloodletting-cupping + western medicine; (e) acupuncture; (f) fire acupuncture + western medicine; (g) fire acupuncture; (h) bloodletting-cupping; (i) acupoint injection + Western medicine; (j) acupoint injection; (k) electroacupuncture + western medicine; (l) acupoint embedding; (n) electroacupuncture; and (o) acupoint embedding + western medicine.

Thirty included studies reported pain scores [23–52] involving 14 treatment regimens, forming a total of 6 closed loops; 12 studies reported overall effectiveness [23, 25, 28–30, 35, 37, 38, 42, 47, 49, 52] involving 9 treatment regimens, forming a total of 5 closed loops; and 14 studies [25, 26, 31, 33, 36, 39, 43–45, 47–49, 51, 52] reported adverse effects involving 11 treatment regimens, with no closed loops formed. The thicker the line between the two, the greater the number of studies between the two measures, the larger the node and the larger the sample size of studies involving this intervention, as shown in Figures 3–5.

#### 3.5.2. Results of Reticulated Meta-Analysis of Pain Scores

**Figure 6:**
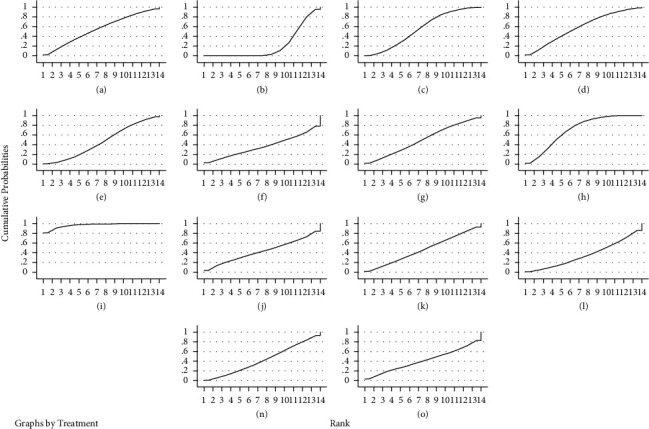
SUCRA ranking of pain scores for different interventions. *Notes*. In the figure, RANK is the horizontal coordinate, indicating the possible ranks, cumulative probabilities is the vertical coordinate, indicating the probability of being at this rank, and the area under the curve is used to represent the overall probability of the treatment measure.

Thirty studies reported pain scores [23–52], with inconsistency tests *P* = 0.992 > 0.05, and local inconsistency tests using the nodal split method with *P* > 0.05, showing good agreement between studies and reticulated meta-analysis under the consistency model. The results showed that acupoint injection + Western medicine was superior to acupuncture, acupuncture + Western medicine, electroacupuncture, bloodletting-cupping and Western medicine, and bloodletting-cupping + Western medicine was superior to Western medicine. The rest of the comparisons between the different treatments were not statistically different, see Table 4. The results of the ranking of pain scores were: acupoint injection + Western medicine (97%) > bloodletting-cupping (70.7%) > bloodletting-cupping + Western medicine (59%) > warm acupuncture (55.7) > acupuncture + Western medicine (55.6%) > fire acupuncture (49.8%) > electroacupuncture + Western medicine (46.6%) > acupuncture (46%) > electroacupuncture (43.4%) > acupoint embedding + Western medicine (42.5%) + (40.8%) > acupuncture (35.8%) > Western medicine (20.9%), as shown in Figure 6 for the SUCRA ranking chart.

#### 3.5.3. Results of the Net Meta-Analysis of Total Efficiency

**Figure 7:**
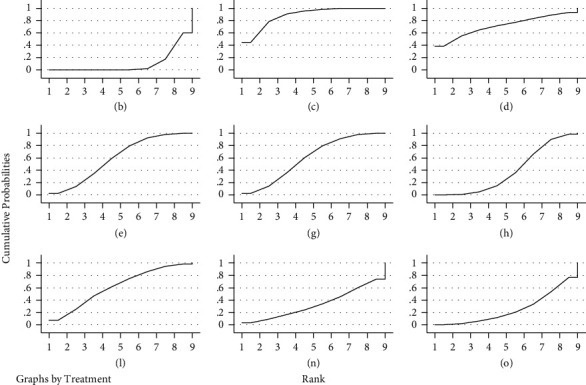
SUCRA ranking of the total effectiveness of different interventions. *Notes*. In the figure, RANK is the horizontal coordinate, indicating the possible ranks; cumulative probabilities is the vertical coordinate, indicating the probability of being at this rank, and the area under the curve is used to represent the overall probability of the treatment measure.

Twelve studies reported overall effective rates [23, 25, 28–30, 35, 37, 38, 42, 47, 49, 52] with inconsistency tests *P* = 0.862 > 0.05 and local inconsistency tests using the nodal split method with *P* > 0.05, showing good agreement between studies and reticulated meta-analysis under the consistency model. The results showed that acupuncture + Western medicine was superior to bloodletting-cupping, bloodletting-cupping + Western medicine and Western medicine, acupuncture was superior to Western medicine, fire acupuncture was superior to Western medicine. The rest of the comparisons between different treatments were not statistically different, see Table 5. The results of the ranking of the total efficiency were: acupuncture + Western medicine (88.3%) > bloodletting-cupping + Western medicine (71.5%) > acupoint embedding (61.9%) > fire acupuncture (60.2%) > acupuncture (60.1%) > bloodletting-cupping (39%) > electroacupuncture (33.5%) > acupoint embedding + Western medicine (25.6%) > Western medicine (10%), see Figure 7 for the SUCRA ranking chart.

#### 3.5.4. Results of the Reticulated Meta-Analysis of Adverse Reactions

**Figure 8:**
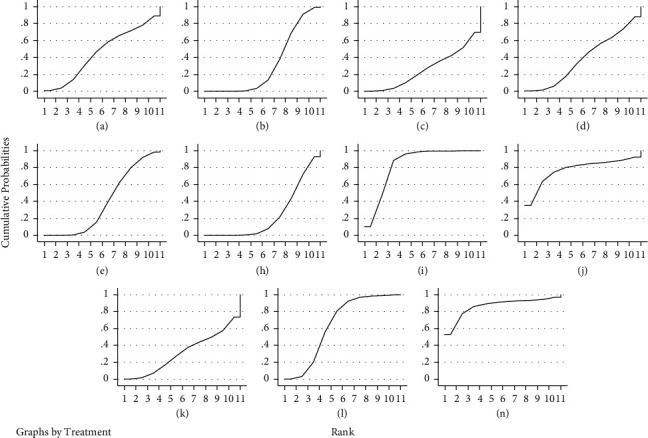
SUCRA ranking of rates of adverse reactions to different interventions. Notes. In the figure, RANK is the horizontal coordinate, indicating the possible ranks, cumulative probabilities is the vertical coordinate, indicating the probability of being at this rank, and the area under the curve is used to represent the overall probability of the treatment measure.

Adverse effects were reported in 14 studies [25, 26, 31, 33, 36, 39, 43–45, 47–49, 51, 52]. As they are all indirect comparisons and do not form a closed loop, no consistency test was required. A mesh meta-analysis was performed under the consistency model. The results showed that acupoint injection + Western medicine was superior to acupuncture, acupuncture + Western medicine, bloodletting-cupping, and Western medicine, and acupoint embedding was superior to Western medicine and bloodletting-cupping. The rest of the comparisons between the different treatments were not statistically different, see Table 6. The results of the ranking of the incidence of adverse reactions (from lowest to highest) were: electroacupuncture (86.7%) > acupoint injection + Western medicine (84%) > acupoint injection (77.5%) > acupoint embedding (64.8%) > warm acupuncture (45.9%) > acupuncture (39.3%) > bloodletting-cupping + Western medicine (38.6%) > electroacupuncture + Western medicine (31.6%) > Western medicine (31.5%) > acupuncture + Western medicine (26.1%) > bloodletting-cupping (24%), see Figure 8 for the SUCRA ranking chart.

#### 3.5.5. Small Sample Effect Estimation

**Figure 9:**
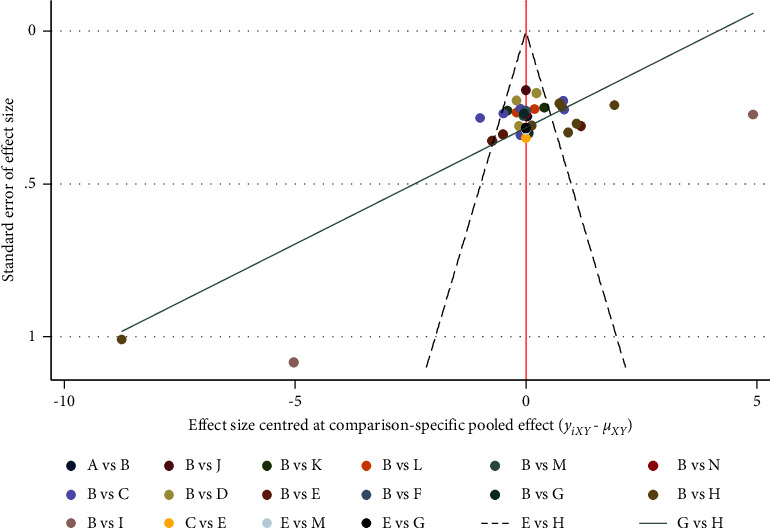
Comparison of pain scores-corrected funnel plot. Notes. (a) Warm acupuncture; (b) Western medicine; (c) acupuncture + Western medicine; (d) bloodletting-cupping + Western medicine; (e) acupuncture; (f) fire acupuncture + Western medicine; (g) fire acupuncture; (h) bloodletting-cupping; (i) acupoint injection + Western medicine; (j) acupoint injection; (k) electroacupuncture + Western medicine; (l) acupoint embedding; (n) electroacupuncture; (o) acupoint embedding + Western medicine.

A comparative-corrected funnel plot of pain scores for the main outcome indicators was assessed by Stata 14.2 software, see Figure 9. The results showed that the funnel plot was not fully symmetrical, suggesting that there may be some publication bias or small sample effect in the study network.

## 4. Discussion

Patients with PHN are often accompanied by prolonged and persistent pain, which triggers other symptoms such as anxiety, depression, and sleep disturbances [53]. Although a variety of treatment options have been proposed, first-line treatment options are still predominant in clinical practice [54], but their efficacy is not entirely reliable [8]. Acupuncture therapy based on meridian theory is widely regarded as potentially beneficial and safe for the treatment of neuropathic pain [55, 56]. The efficacy and safety of acupuncture alone or in combination with Western medicine in the treatment of PHN has been clinically proven, and the selection of the optimal combination has become the focus of current research.

This study evaluated the effects of acupuncture-related therapies alone or in combination with Western medicine on pain scores, overall effectiveness, and adverse reaction rates in patients with PHN. The results of the study showed that acupoint injection + Western medicine was superior to acupuncture, acupuncture + Western medicine, electroacupuncture, bloodletting-cupping, and Western medicine, and bloodletting-cupping + Western medicine was superior to Western medicine in terms of improving pain scores. The results of the probability ranking showed that acupoint injection + Western medicine > bloodletting-cupping > bloodletting-cupping + Western medicine > warm acupuncture > acupuncture + Western medicine > fire acupuncture > electroacupuncture + Western medicine > acupuncture > electroacupuncture > acupoint embedding + Western medicine > fire acupuncture + Western medicine > acupoint embedding > Western medicine. In terms of total efficiency, acupuncture + Western medicine was superior to bloodletting-cupping, bloodletting-cupping + Western medicine and Western medicine, acupuncture was superior to Western medicine and fire acupuncture was superior to Western medicine. The results of the probability ranking showed that acupuncture + Western medicine > bloodletting-cupping + Western medicine > acupoint embedding > fire acupuncture > acupuncture > bloodletting-cupping > electroacupuncture > acupoint embedding + Western medicine > Western medicine. In terms of adverse reaction rates, acupoint injection + Western medicine was superior to acupuncture, acupuncture + Western medicine, bloodletting-cupping and Western medicine, and acupoint embedding was superior to Western medicine and bloodletting-cupping. The results of the probability ranking showed that electroacupuncture > acupoint injection + Western medicine > acupoint injection > acupoint embedding > warm acupuncture > acupuncture > bloodletting-cupping + Western medicine > electroacupuncture + Western medicine > Western medicine > acupuncture + Western medicine > bloodletting-cupping. The analysis of the above indicators showed that Western medicine ranked low in terms of improvement in pain, overall effectiveness, and adverse reaction rate, which could be seen as a complementary and alternative option to first-line treatment options. Although the ranking of efficacy varied between indicators, the rankings of acupoint injection + Western medicine, bloodletting-cupping, bloodletting-cupping + Western medicine, and acupuncture + Western medicine were ranked at the top, and the combined results of the pain score and the ranking of the total effective rate showed that acupoint injection + Western medicine and bloodletting-cupping + Western medicine were outstanding in the treatment of PHN. Combining the results of the direct meta-analysis followed by the reticulated meta-analysis, we found no significant differences in adverse reaction rates between the treatment regimens. Considering the moderate quality of the included studies, the selection needs to be clinically justified in relation to the characteristics of the patients' conditions, and the probability ranking results are for clinicians' reference only.

### 4.1. Limitations of this study

(i) Some of the studies did not specifically describe the randomisation method, allocation concealment, and blinding, which affected the efficacy of testing the results. (ii) The small sample size of the included studies may have limited the accuracy of the results of this study. (iii) The type and dosage of anti-rheumatic drugs, the selection of acupuncture points for acupuncture-related therapies, and the duration of treatment vary in the included literature, which may increase clinical heterogeneity. (iv) There was some publication bias and a small sample effect in the included studies, which affects the reliability of the findings.

## 5. Conclusion

In summary, after a comprehensive comparison of the outcome indicators of 14 different treatment regimens, acupoint injection + Western medicine and bloodletting-cupping + Western medicine were considered to be the best combination regimens for the treatment of PHN. The appropriate treatment modality should be selected in clinical practice in the context of the actual situation. Due to the limitations of the study, the above conclusions still need to be validated by further multicentre, large-sample prospective randomised controlled clinical trials.

## Figures and Tables

**Table 1:** PubMed database search strategy.

Number	Search terms
#1	Acupuncture [MeSH]
#2	Acupuncture [title/abstract]
#3	Cupping [title/abstract]
#4	Electroacupuncture [title/abstract]
#5	Warm needle [title/abstract]
#6	Fire needle [title/abstract]
#7	Blood-letting [title/abstract]
#8	Moxibustion [MeSH]
#9	Moxibustion [title/abstract]
#10	Auricular application pressure [title/abstract]
#11	Auricular needle [title/abstract]
#12	Acupoint embedding [title/abstract]
#13	Acupoint injection [title/abstract]
#14	#1 OR #2 OR #3 OR #4 OR #5 OR #6 OR #7 OR #8 OR #9 OR #10 OR #11 OR #12 OR #13
#15	Neuralgia, postherpetic [MeSH]
#16	Postherpetic neuralgia [title/abstract]
#17	#15 OR #16
#18	#14 AND #18

**Table 2:** Basic characteristics of included studies.

Included studies	Random method	Sample (T/C)	Gender (M/F)	Age (years)	Course (months)
Huang [31]	Random number table	30/30	T: 14/16	T: 65.20 ± 9.93	T: 4.03 ± 6.34
			C: 13/17	C:66.07 ± 9.68	C: 3.90 ± 4.80
Liu [26]	Unclear	38/34	T: 18/20	T: 68.32 ± 9.74	T: 1.58 ± 1.18
			C: 13/21	C: 69.21 ± 9.21	C: 1.53 ± 1.02
Zhang [50]	Unclear	63/63	T: 35/28	T: 62.70 ± 6.83	T: 5.13 ± 0.81
			C: 34/29	C: 62.54 ± 6.78	C: 5.10 ± 0.79
Deng [29]	Random number table	43/43	T: 31/12	T: 55.40 ± 7.93	T: 22.17 ± 4.44
			C: 28/15	C: 21.58 ± 4.13	C: 21.58 ± 4.13
Zhai [30]	Computer generated random numbers	40/40	T: 17/23	T: 63.7 ± 6.8	T: 8.50 ± 3.46
			C: 19/21	C: 62.4 ± 7.6	C: 8.43 ± 4.36
Ding [51]	Random number table	57/58	T: 25/32	T: 59.33 ± 6.25	T: 3.71 ± 0.49
			C: 27/31	C: 62.45 ± 9.23	C: 3.49 ± 0.63
Wang H 2014	Unclear	21/21/21	Unclear	Unclear	Unclear
Chen [27]	Unclear	27/27	T: 16/11	T: 62.18 ± 7.98	Unclear
			C: 14/13	C: 63.25 ± 7.49	
Zhou YN 2018	Random number table	30/30	T: 14/16	T: 52.78 ± 8.12	T: 6.85 ± 4.48
			C: 12/18	C: 53.34 ± 7.60	C: 6.62 ± 4.13
Zhang [25]	Random number table	20/20/20	T1: 8/12	T1: 60 ± 7	T1: 1.62 ± 0.54
		/20	T2: 13/7	T2: 60 ± 7	T2: 1.76 ± 0.43
			T3: 12/8	T3: 61 ± 7	T3: 1.59 ± 0.68
			C: 9/11	C: 60 ± 8	C: 1.65 ± 0.51
Li [33]	Unclear	30/31	T: 13/17	T: 53 ± 12	T: 1.5 ± 1.1
			C: 14/17	C: 56 ± 10	C: 1.7 ± 0.9
Liu [38]	Unclear	31/31	T: 14/17	T: 59.90 ± 9.07	T: 55.22 ± 15.01 (days)
			C: 15/16	C: 60.25 ± 8.79	C: 57.58 ± 15.09 (days)
Liu B 2020	Computer generated random numbers	38/27	T: 21/17	T: 64.5 ± 3.5	T: 4.1 ± 1.2
			C: 16/11	C: 67.5 ± 3.8	C: 3.3 ± 0.6
Wang [35]	Random number table	35/35	T: 16/19	T: 57.8	T: 62.73 ± 10.15 (days)
			C: 17/18	C: 58.6	C: 61.82 ± 9.48 (days)
Liu [37]	Order of consultation	40/40	T: 22/18	T: 50.25 ± 10.17	Unclear
			C: 15/15	C: 50.18 ± 10.38	
Liu [39]	Random number table	62/58	T: 37/25	T: 26.30 ± 7.66	Unclear
			C: 35/23	C: 26.66 ± 7.28	
Qing L 2019	Random number table	25/25	Unclear	Unclear	Unclear
			
Tian [41]	Random number table	34/34	T: 14/18	T: 61	T: 4
			C: 16/16	C:61	C: 4
Liu [42]	Order of consultation	35/33	T: 18/17	T: 49	Unclear
			C: 19/14	C: 52	
Wang [43]	Unclear	41/36	Unclear	Unclear	Unclear
			
Xie YJ 2013	Random number table	40/40	T: 21/19	T: 65.3 ± 10.4	Unclear
			C: 18/22	C: 65.8 ± 10.8	
Li LP 2019	Random number table	30/29	T: 16/14	T: 59 ± 11	T: 1.1 ± 0.15 (years)
			C: 14/15	C: 57 ± 13	C: 1.2 ± 0.13 (years)
Yang [46]	Computer generated random numbers	34/34	T: 22/12	T: 56.8 ± 14.9	T: 4.9 ± 3.8
			C: 23/11	C: 54.3 ± 12.4	C: 5.6 ± 3.4
Chen [28]	Random number table	30/28	T: 15/15	T: 55.17 ± 10.88	T: 6.23 ± 1.61
			C: 13/15	C: 52.93 ± 10.4	C: 6.41 ± 1.92
Wang [47]	Unclear	40/40	T: 21/19	T: 59.43 ± 5.27	T: 5.73 ± 1.16
			C: 20/20	C: 60.13 ± 4.29	C: 5.98 ± 1.03
Zhang [48]	Random number table	45/45	T: 12/33	T: 48.49 ± 4.29	Unclear
			C: 11/34	C: 46.39 ± 6.38	
Zhang [23]	Random number table	20/20/19	T1: 5/15	T1: 62.55 ± 7.48	Unclear
			T2: 9/11	T2: 64.2 ± 10.81	
			C: 11/8	C: 66.79 ± 11.25	
Zhang [49]	Random number table	31/30	T: 16/15	T: 61.69 ± 8.43	T: 7.43 ± 1.49
			C: 17/13	C: 61.42 ± 7.96	C: 7.68 ± 1.52
Lin [36]	Random number table	32/31	T: 12/18	T: 54.63 ± 4.57	T: 7.33 ± 6.24
			C: 13/17	C: 54.03 ± 5.5	C: 7.57 ± 5.89
Zhong [52]	Random number table	33/33	T: 16/17	T: 60.03 ± 3.9	T: 3.61 ± 1
			C: 15/18	C: 59.55 ± 5.36	C: 3.58 ± 1

Notes. T, Treatment group; C, Control group; M, Male; F, Female.

**Table 3:** Characteristics of the included study interventions.

Inclusion of literature	Type of research	Interventions		Treatment course (days)	Outcome indicators
		Treatment group	Control group		
Huang [31]	Two-armed	Electroacupuncture: EX-B2, LR1, LR2, ST44, ashi point, six times a week.	Pregabalin capsules: 2 times/day, 75 mg/dose.	14	①②
Liu [26]	Two-armed	Warm acupuncture: Ashi point, once every two days.	Gabapentin capsules: Day 1, 100 mg/dose, 3 times/day; day 2, 200 mg/dose, 3 times/day; day 3, 300 mg/dose, 3 times/day.	10	①②
Zhang [50]	Two-armed	Bloodletting-cupping: Ashi point, once every two days. Pregabalin capsules: 2 times/day, 75 mg/dose.	Pregabalin capsules: 2 times/day, 75 mg/dose.	14	①
Deng [29]	Two-armed	Acupuncture: Ashi point, once a day. Gabapentin capsules: 300 mg/dose, 3 times/day; mecobalamin tablets: 0.5 mg/dose, 3 times/day.	Gabapentin capsules: 300 mg/dose, 3 times/day; mecobalamin tablets: 0.5 mg/dose, 3 times/day.	14	①③
Zhai [30]	Two-armed	Acupuncture: GB36, GB35, LI7, ST34, SI6, BL63, BL59, TE 7	Pregabalin capsules: 2 times/day, 75 mg/dose.	35	①③
Ding [51]	Two-armed	Bloodletting-cupping: Ashi point, 3 times a week. Gabapentin capsules: 300 mg/dose, 1 time/day; mecobalamin tablets: 0.5 mg/dose, 3 times/day.	Gabapentin capsules: 300 mg/dose, 1 time/day; mecobalamin tablets: 0.5 mg/dose, 3 times/day.	28	①②
Wang H 2014	Three-armed	①Acupuncture: EX-B2, once a day 1, 1 ②Acupuncture: EX-B2, once a day. Gabapentin capsules: 150 mg/dose on day 1, 1 time/day, 150 mg/dose on day 2, 2 times/day, 150 mg/dose on day 3, 3 times/day, with subsequent increases of 150 mg/day every 1 to 2 days (maximum dose 2,400 mg/day)	Gabapentin capsules: 150 mg/dose on day 1, 1 time/day, 150 mg/dose on day 2, 2 times/day, 150 mg/dose on day 3, 3 times/day, with subsequent increases of 150 mg/day every 1 to 2 days (maximum dose 2,400 mg/day)	14	①
Chen [27]	Two-armed	Warm acupuncture: EX-B2, LR3, GB41, ashi point, once every two days.	Carbamazepine: 100 mg/dose, 2 times/day.	28	①
Zhou YN 2018	Two-armed	Fire acupuncture: Heart and diaphragm, 3 times a week	Pregabalin capsules: 3 times/day, 50 mg/dose.	10	①
Zhang [25]	Four-armed	①Fire acupuncture: Ashi point, once every two days. ②Acupuncture: Ashi point, once a day. ③Bloodletting-cupping: Ashi point, 1 time every other day.	Pregabalin capsules: 2 times/day, 150 mg/dose.	30	①②③
Li [33]	Two-armed	Acupoint injection: EX-B2, once a week. Gabapentin capsules: 200–900 mg/day.	Gabapentin capsules: 200–900 mg/day.	28	①②
Liu [38]	Two-armed	Acupuncture point embedding: DU10, once every two days.	Carbamazepine: 100 mg/dose, 3 times/day.	28	①③
Liu B 2020	Two-armed	Acupuncture: EX-B2, BL17, BL18, ST36, LR3, GB34, KI3. Pregabalin capsules: 2 times/day, 150 mg/dose.	Pregabalin capsules: 2 times/day, 150 mg/dose.	14	①
Wang [35]	Two-armed	Stabbing cupping: Ashi point, once every two days.	Gabapentin capsules: 300 mg once daily on day 1, 300 mg twice daily on day 2 and 300 mg three times daily after day 3.	16	①③
Liu [37]	Two-armed	Bloodletting-cupping: Ashi point, once time every 5 days.	Pregabalin capsules: 2 times/day, 75 mg/dose.	30	①③
Liu [39]	Two-armed	Acupoint injection: EX-B2, once every two days.	Gabapentin capsules: 300 mg once daily on day 1, 300 mg twice daily on day 2 and 300 mg three times daily after day 3.	28	①②
Qing L 2019	Two-armed	Bloodletting-cupping: Ashi point, 1 time daily for the first 3 days, 1 time every other day from the 4th day onwards.	Gabapentin capsules: 300 mg/dose, 3 times/day; mecobalamin tablets: 0.5 mg/dose, 3 times/day.	30	①
Tian [41]	Two-armed	Bloodletting-cupping: Ashi point, once every two days.	Pregabalin capsules: 2 times/day, 150 mg/dose.	16	①
Liu [42]	Two-armed	Bloodletting-cupping: Ashi point, once every two days.	Flupirtine melitrexin: 10.5 mg/dose, 2 times/day; mecobalamin tablets: 0.5 mg/dose, 3 times/day.	28	①③
Wang [43]	Two-armed	Electroacupuncture: EX-B2, once a day. Pregabalin capsules: 150–600 mg/day in 2 or 3 oral doses.	Pregabalin capsules: 150–600 mg/day in 2 or 3 oral doses.	14	①②
Xie YJ 2013	Two-armed	Acupuncture: Ashi point, once every two days; gabapentin capsules: 300 mg orally on day 1, increasing to 600, 900 mg on days 2–3, 1200 mg/day on days 4–6, 1500 mg on day 7 and 1800 mg/day on days 8–42.	Gabapentin capsules: 300 mg orally on day 1, increasing to 600, 900 mg on days 2–3, 1200 mg/day on days 4–6, 1500 mg on day 7 and 1800 mg/day on days 8–42.	42	①②
Li LP 2019	Two-armed	Acupoint injection: Ashi point, once a week; gabapentin capsules: 300 mg orally on day 1, increasing to 900 mg on day 3, gradually increasing to 1800 mg/day.	Gabapentin capsules: 300 mg orally on day 1, increasing to 900 mg on day 3, gradually increasing to 1800 mg/day.	21	①②
Yang [46]	Two-armed	Electroacupuncture: Jiaji and ashi points, once a day.	Gabapentin capsules: 300 mg once daily on day 1, 300 mg twice daily on day 2 and 300 mg three times daily after day 3.	28	①
Chen [28]	Two-armed	Acupuncture point embedding: EX-B2, once every fortnight. Pregabalin capsules: 2 times/day, 75 mg/dose.	Pregabalin capsules: 2 times/day, 75 mg/dose.	28	①③
Wang [47]	Two-armed	Acupuncture: EX-B2, ashi, ST36. Gabapentin capsules: 300 mg once daily on day 1, 300 mg twice daily on day 2 and 300 mg three times daily after day 3.	Gabapentin capsules: 300 mg once daily on day 1, 300 mg twice daily on day 2 and 300 mg three times daily after day 3.	42	①②③
Zhang [48]	Two-armed	Bloodletting-cupping: Ashi point, once every two days. Pregabalin capsules: 2 times/day, 75 mg/dose.	Pregabalin capsules: 2 times/day, 75 mg/dose.	14	①②
Zhang [23]	Three-armed	①Acupuncture: TE6, GB34, ST36, ashi points, once a day. ②Electroacupuncture: TE6, GB34, ST36, ashi points, once a day.	Gabapentin capsules: 300 mg once daily on day 1, 300 mg twice daily on day 2 and 300 mg three times daily after day 3.	20	①③
Zhang [49]	Two-armed	Bloodletting-cupping: Ashi point, once every two days. Pregabalin capsules: 2 times/day, 150 mg/dose.	Pregabalin capsules: 2 times/day, 150 mg/dose.	30	①②③
Lin [36]	Two-armed	Acupoint embedding: EX-B2, ashi points, once every 2 weeks.	Carbamazepine: 100 mg/dose, 2 times/day.	56	①②
Zhong [52]	Two-armed	Fire acupuncture: EX-B2, ashi points, once every two days.	Pregabalin capsules: 2 times/day, 75 mg/dose.	28	①②③

Notes. ① Pain scores; ② Adverse reactions; ③ Total efficiency.

**Table 4:** Results of the reticulated meta-analysis of pain scores.

I	3.23 (−0.49, 6.95)	3.77 (−0.44, 7.98)	3.86 (−0.66, 8.38)	3.97 (0.19, 7.75)	4.18 (−0.24, 8.59)	4.41 (−0.18, 9.00)	4.40 (0.35, 8.45)	4.58 (−0.98, 10.15)	4.63 (0.15, 9.12)	4.81 (−0.77, 10.38)	5.16 (−0.41, 10.74)	4.98 (0.39, 9.57)	5.50 (2.18, 8.83)

−3.23 (−6.95, 0.49)	H	0.54 (−2.54, 3.62)	0.63 (−2.86, 4.11)	0.74 (−1.70, 3.18)	0.95 (−2.20, 4.10)	1.18 (−2.40, 4.76)	1.17 (−1.53, 3.87)	1.35 (−3.41, 6.12)	1.40 (−2.01, 4.81)	1.58 (−3.21, 6.36)	1.93 (−2.85, 6.71)	1.75 (−1.83, 5.33)	2.28 (0.60, 3.95)

−3.77 (−7.98, 0.44)	−0.54 (−3.62, 2.54)	D	0.09 (−3.91, 4.09)	0.20 (−2.94, 3.35)	0.41 (−3.47, 4.29)	0.65 (−3.44, 4.73)	0.63 (−2.84, 4.10)	0.82 (−4.34, 5.97)	0.86 (−3.10, 4.83)	1.04 (−4.13, 6.21)	1.39 (−3.77, 6.56)	1.21 (−2.87, 5.29)	1.74 (−0.84, 4.32)

−3.86 (−8.38, 0.66)	−0.63 (−4.11, 2.86)	−0.09 (−4.09, 3.91)	A	0.11 (−3.43, 3.66)	0.32 (−3.89, 4.53)	0.56 (−3.84, 4.95)	0.54 (−3.29, 4.38)	0.73 (−4.68, 6.13)	0.77 (−3.51, 5.06)	0.95 (−4.47, 6.37)	1.30 (−4.11, 6.72)	1.12 (−3.28, 5.52)	1.65 (−1.42, 4.71)

−**3.97 (**−**7.75**, −**0.19)**	−0.74 (−3.18, 1.70)	−0.20 (−3.35, 2.94)	−0.11 (−3.66, 3.43)	C	0.20 (−3.17, 3.58)	0.44 (−3.20, 4.08)	0.43 (−2.31, 3.17)	0.61 (−4.20, 5.42)	0.66 (−2.80, 4.12)	0.83 (−3.99, 5.66)	1.19 (−3.63, 6.01)	1.01 (−2.63, 4.64)	1.53 (−0.26, 3.33)

−4.18 (−8.59, 0.24)	−0.95 (−4.10, 2.20)	−0.41 (−4.29, 3.47)	−0.32 (−4.53, 3.89)	−0.20 (−3.58, 3.17)	G	0.24 (−4.05, 4.53)	0.23 (−3.16, 3.61)	0.41 (−4.91, 5.73)	0.46 (−3.64, 4.56)	0.63 (−4.70, 5.96)	0.99 (−4.35, 6.32)	0.80 (−3.49, 5.09)	1.33 (−1.57, 4.23)

−4.41 (−9.00, 0.18)	−1.18 (−4.76, 2.40)	−0.65 (−4.73, 3.44)	−0.56 (−4.95, 3.84)	−0.44 (−4.08, 3.20)	−0.24 (−4.53, 4.05)	K	−0.01 (−3.93, 3.91)	0.17 (−5.30, 5.64)	0.22 (−4.15, 4.59)	0.39 (−5.09, 5.87)	0.75 (−4.73, 6.23)	0.56 (−3.91, 5.04)	1.09 (−2.07, 4.26)

−**4.40 (**−**8.45**, −**0.35)**	−1.17 (−3.87, 1.53)	−0.63 (−4.10, 2.84)	−0.54 (−4.38, 3.29)	−0.43 (−3.17, 2.31)	−0.23 (−3.61, 3.16)	0.01 (−3.91, 3.93)	E	0.18 (−4.85, 5.21)	0.23 (−3.14, 3.60)	0.40 (−4.64, 5.45)	0.76 (−4.28, 5.80)	0.58 (−3.35, 4.50)	1.10 (−1.22, 3.42)

−4.58 (−10.15, 0.98)	−1.35 (−6.12, 3.41)	−0.82 (−5.97, 4.34)	−0.73 (−6.13, 4.68)	−0.61 (−5.42, 4.20)	−0.41 (−5.73, 4.91)	−0.17 (−5.64, 5.30)	−0.18 (−5.21, 4.85)	J	0.05 (−5.33, 5.43)	0.22 (−6.10, 6.54)	0.58 (−5.74, 6.90)	0.39 (−5.08, 5.86)	0.92 (−3.54, 5.38)

−**4.63 (**−**9.12**, −**0.15)**	−1.40 (−4.81, 2.01)	−0.86 (−4.83, 3.10)	−0.77 (−5.06, 3.51)	−0.66 (−4.12, 2.80)	−0.46 (−4.56, 3.64)	−0.22 (−4.59, 4.15)	−0.23 (−3.60, 3.14)	−0.05 (−5.43, 5.33)	N	0.17 (−5.22, 5.57)	0.53 (−4.86, 5.92)	0.34 (−4.02, 4.71)	0.87 (−2.14, 3.88)

−4.81 (−10.38, 0.77)	−1.58 (−6.36, 3.21)	−1.04 (−6.21, 4.13)	−0.95 (−6.37, 4.47)	−0.83 (−5.66, 3.99)	−0.63 (−5.96, 4.70)	−0.39 (−5.87, 5.09)	−0.40 (−5.45, 4.64)	−0.22 (−6.54, 6.10)	−0.17 (−5.57, 5.22)	O	0.36 (−5.97, 6.69)	0.17 (−5.31, 5.65)	0.70 (−3.78, 5.18)

−5.16 (−10.74, 0.41)	−1.93 (−6.71, 2.85)	−1.39 (−6.56, 3.77)	−1.30 (−6.72, 4.11)	−1.19 (−6.01, 3.63)	−0.99 (−6.32, 4.35)	−0.75 (−6.23, 4.73)	−0.76 (−5.80, 4.28)	−0.58 (−6.90, 5.74)	−0.53 (−5.92, 4.86)	−0.36 (−6.69, 5.97)	F	−0.18 (−5.66, 5.30)	0.34 (−4.13, 4.82)

−**4.98 (**−**9.57**, −**0.39)**	−1.75 (−5.33, 1.83)	−1.21 (−5.29, 2.87)	−1.12 (−5.52, 3.28)	−1.01 (−4.64, 2.63)	−0.80 (−5.09, 3.49)	−0.56 (−5.04, 3.91)	−0.58 (−4.50, 3.35)	−0.39 (−5.86, 5.08)	−0.34 (−4.71, 4.02)	−0.17 (−5.65, 5.31)	0.18 (−5.30, 5.66)	L	0.53 (−2.64, 3.69)

−**5.50 (**−**8.83**, −**2.18)**	−**2.28 (**−**3.95**, −**0.60)**	−1.74 (−4.32, 0.84)	−1.65 (−4.71, 1.42)	−1.53 (−3.33, 0.26)	−1.33 (−4.23, 1.57)	−1.09 (−4.26, 2.07)	−1.10 (−3.42, 1.22)	−0.92 (−5.38, 3.54)	−0.87 (−3.88, 2.14)	−0.70 (−5.18, 3.78)	−0.34 (−4.82, 4.13)	−0.53 (−3.69, 2.64)	B

Notes. The above data represented the confidence interval. The bold font indicated that there was a statistically significant difference between the two treatments. A, warm acupuncture; B, Western medicine; C, acupuncture + Western medicine; D, bloodletting-cupping + Western medicine; E, acupuncture; F, fire acupuncture + Western medicine; G, fire acupuncture; H, bloodletting-cupping; I: acupoint injection + Western medicine; J, acupoint injection; K, electroacupuncture + Western medicine; L, acupoint embedding; N, electroacupuncture; O, acupoint embedding + Western medicine.

**Table 5:** Results of the reticulated meta-analysis of total efficiency.

C	−0.03 (−0.43, 0.36)	−0.11 (−0.34, 0.12)	−0.13 (−0.33, 0.08)	−0.13 (−0.33, 0.08)	−0.19 (−0.37, −0.01)	−0.22 (−0.52, 0.08)	−0.25 (−0.48, −0.02)	−0.29 (−0.44, −0.14)

0.03 (−0.36, 0.43)	D	−0.08 (−0.49, 0.33)	−0.09 (−0.49, 0.30)	−0.09 (−0.49, 0.31)	−0.16 (−0.54, 0.23)	−0.19 (−0.64, 0.27)	−0.21 (−0.62, 0.19)	−0.25 (−0.62, 0.11)

0.11 (−0.12, 0.34)	0.08 (−0.33, 0.49)	L	−0.01 (−0.24, 0.21)	−0.01 (−0.24, 0.22)	−0.08 (−0.28, 0.13)	−0.11 (−0.43, 0.21)	−0.14 (−0.38, 0.11)	−0.18 (−0.35, 0.00)

0.13 (−0.08, 0.33)	0.09 (−0.30, 0.49)	0.01 (−0.21, 0.24)	G	0.00 (−0.14, 0.14)	−0.07 (−0.21, 0.08)	−0.09 (−0.36, 0.18)	−0.12 (−0.34, 0.10)	−0.16 (−0.30, −0.02)

0.13 (−0.08, 0.33)	0.09 (−0.31, 0.49)	0.01 (−0.22, 0.24)	−0.00 (−0.14, 0.14)	E	−0.07 (−0.21, 0.08)	−0.09 (−0.33, 0.14)	−0.12 (−0.35, 0.10)	−0.16 (−0.31, −0.02)

**0.19 (0.01**, **0.37)**	0.16 (−0.23, 0.54)	0.08 (−0.13, 0.28)	0.07 (−0.08, 0.21)	0.07 (−0.08, 0.21)	H	−0.03 (−0.30, 0.24)	−0.06 (−0.26, 0.14)	−0.10 (−0.20, 0.00)

0.22 (−0.08, 0.52)	0.19 (−0.27, 0.64)	0.11 (−0.21, 0.43)	0.09 (−0.18, 0.36)	0.09 (−0.14, 0.33)	0.03 (−0.24, 0.30)	N	−0.03 (−0.35, 0.29)	−0.07 (−0.33, 0.20)

**0.25 (0.02**, **0.48)**	0.21 (−0.19, 0.62)	0.14 (−0.11, 0.38)	0.12 (−0.10, 0.34)	0.12 (−0.10, 0.35)	0.06 (−0.14, 0.26)	0.03 (−0.29, 0.35)	O	−0.04 (−0.21, 0.13)

**0.29 (0.14**, **0.44)**	0.25 (−0.11, 0.62)	0.18 (−0.00, 0.35)	**0.16 (0.02**, **0.30)**	**0.16 (0.02**, **0.31)**	0.10 (−0.00, 0.20)	0.07 (−0.20, 0.33)	0.04 (−0.13, 0.21)	B

Notes. The above data represented the confidence interval. The bold font indicated that there was a statistically significant difference between the two treatments. B, Western medicine; C, acupuncture + Western medicine; D, bloodletting-cupping + Western medicine; E, acupuncture; G, fire acupuncture; H, bloodletting-cupping; L, acupoint embedding; N, electroacupuncture; O, acupoint embedding + Western medicine.

**Table 6:** Results of the reticulated meta-analysis of adverse reactions.

N	0.97 (−2.03, 3.97)	0.52 (−3.65, 4.69)	1.69 (−1.24, 4.61)	1.97 (−1.09, 5.04)	2.12 (−0.77, 5.01)	2.11 (−0.89, 5.10)	2.25 (−0.83, 5.33)	2.20 (−0.68, 5.08)	2.34 (−0.70, 5.39)	2.25 (−0.63, 5.14)

−0.97 (−3.97, 2.03)	I	−0.45 (−3.58, 2.68)	0.71 (−0.28, 1.71)	1.00 (−0.35, 2.35)	1.15 (0.25, 2.04)	1.14 (−0.05, 2.32)	1.28 (−0.11, 2.67)	1.23 (0.37, 2.08)	1.37 (0.07, 2.68)	1.28 (0.41, 2.15)

−0.52 (−4.69, 3.65)	0.45 (−2.68, 3.58)	J	1.16 (−1.89, 4.22)	1.45 (−1.74, 4.64)	1.59 (−1.43, 4.62)	1.59 (−1.54, 4.71)	1.73 (−1.48, 4.94)	1.68 (−1.34, 4.69)	1.82 (−1.35, 5.00)	1.73 (−1.29, 4.75)

−1.69 (−4.61, 1.24)	−0.71 (−1.71, 0.28)	−1.16 (−4.22, 1.89)	L	0.29 (−0.88, 1.45)	0.43 (−0.14, 1.01)	0.42 (−0.54, 1.39)	0.56 (−0.65, 1.77)	0.51 (0.00, 1.02)	0.66 (−0.45, 1.77)	0.57 (0.03, 1.10)

−1.97 (−5.04, 1.09)	−1.00 (−2.35, 0.35)	−1.45 (−4.64, 1.74)	−0.29 (−1.45, 0.88)	A	0.14 (−0.94, 1.23)	0.14 (−1.20, 1.47)	0.28 (−1.24, 1.79)	0.22 (−0.82, 1.27)	0.37 (−1.07, 1.81)	0.28 (−0.78, 1.34)

−2.12 (−5.01, 0.77)	−**1.15 (**−**2.04**, −**0.25)**	−1.59 (−4.62, 1.43)	−0.43 (−1.01, 0.14)	−0.14 (−1.23, 0.94)	E	−0.01 (−0.88, 0.86)	0.13 (−1.00, 1.27)	0.08 (−0.19, 0.35)	0.23 (−0.80, 1.25)	0.14 (−0.19, 0.46)

−2.11 (−5.10, 0.89)	−1.14 (−2.32, 0.05)	−1.59 (−4.71, 1.54)	−0.42 (−1.39, 0.54)	−0.14 (−1.47, 1.20)	0.01 (−0.86, 0.88)	D	0.14 (−1.23, 1.51)	0.09 (−0.74, 0.91)	0.23 (−1.05, 1.52)	0.15 (−0.70, 0.99)

−2.25 (−5.33, 0.83)	−1.28 (−2.67, 0.11)	−1.73 (−4.94, 1.48)	−0.56 (−1.77, 0.65)	−0.28 (−1.79, 1.24)	−0.13 (−1.27, 1.00)	−0.14 (−1.51, 1.23)	K	−0.05 (−1.15, 1.05)	0.09 (−1.38, 1.57)	0.01 (−1.11, 1.12)

−2.20 (−5.08, 0.68)	−**1.23 (**−**2.08**, −**0.37)**	−1.68 (−4.69, 1.34)	−**0.51 (**−**1.02**, −**0.00)**	−0.22 (−1.27, 0.82)	−0.08 (−0.35, 0.19)	−0.09 (−0.91, 0.74)	0.05 (−1.05, 1.15)	B	0.15 (−0.84, 1.14)	0.06 (−0.12, 0.23)

−2.34 (−5.39, 0.70)	−**1.37 (**−**2.68**, −**0.07)**	−1.82 (−5.00, 1.35)	−0.66 (−1.77, 0.45)	−0.37 (−1.81, 1.07)	−0.23 (−1.25, 0.80)	−0.23 (−1.52, 1.05)	−0.09 (−1.57, 1.38)	−0.15 (−1.14, 0.84)	C	−0.09 (−1.09, 0.92)

−2.25 (−5.14, 0.63)	−**1.28 (**−**2.15**, −**0.41)**	−1.73 (−4.75, 1.29)	−**0.57 (**−**1.10**, −**0.03)**	−0.28 (−1.34, 0.78)	−0.14 (−0.46, 0.19)	−0.15 (−0.99, 0.70)	−0.01 (−1.12, 1.11)	−0.06 (−0.23, 0.12)	0.09 (−0.92, 1.09)	H

Notes. The above data represented the confidence interval. The bold font indicated that there was a statistically significant difference between the two treatments. A, warm acupuncture; B, Western medicine; C, acupuncture + Western medicine; D, bloodletting-cupping + Western medicine; E, acupuncture; H, bloodletting-cupping; I: acupoint injection + Western medicine; J, acupoint injection; K, electroacupuncture + Western medicine; L, acupoint embedding; N, electroacupuncture.

## Data Availability

The data used to support the findings of this study are included with in the article and the supplementary information files.

## Abbreviations

PHN: Postherpetic neuralgia
RCTs: Randomised controlled trials
HZ: Herpes zoster
TCAs: Tricyclic antidepressants
NMA: Network meta-analysis
VAS: Visual analogue scale
CNKI: China knowledge network
SMD: Standard mean difference
RR: Relative risk
SUCRA: Surface under the cumulative ranking.

## Supplementary Materials

Supplementary Materials Table S1: the PRISMA checklist. Table S2: Direct comparison of meta-analysis results. Figure S1∼S10: Sensitivity analysis of pain scores. Figure S11∼S16: Sensitivity analysis of total efficiency. Figure S17∼S19: Sensitivity analysis of adverse reactions.
