# Supplementary material for: Comparison of Efficacy of Acupuncture-Related Therapy in the Treatment of Postherpetic Neuralgia: A Network Meta-Analysis of Randomized Controlled Trials
Source: Evid Based Complement Alternat Med. 2022 Oct 14;2022:3975389. doi: 10.1155/2022/3975389 (PMC9586726; doi:10.1155/2022/3975389)
Supplement: Supplementary Materials — Table S1: the PRISMA checklist. Table S2: Direct comparison of meta-analysis results. Figure S1∼S10: Sensitivity analysis of pain scores. Figure S11∼S16: Sensitivity analysis of total efficiency. Figure S17∼S19: Sensitivity analysis of adverse reactions. [file 3975389.f1.zip › Sensitivity analysis figures.docx]

**Sensitivity analysis of pain scores figure S1～S10**

Figure S1 electroacupuncture vs Western medicine

Figure S2 warm acupuncture vs Western medicine

Figure S3 bloodletting-cupping + Western medicine vs Western medicine

Figure S4 acupuncture + Western medicine vs Western medicine

Figure S5 acupuncture vs Western medicine

Figure S6 fire acupuncture vs Western medicine

Figure S7 bloodletting-cupping vs Western medicine

Figure S8 acupoint injection + Western medicine vs Western medicine

Figure S9 acupoint embedding vs Western medicine

Figure S10 electroacupuncture + Western medicine vs Western medicine

**Sensitivity analysis of total efficiency figure S11～S16**

Figure S11 acupuncture + Western medicine vs Western medicine

Figure S12 fire acupuncture vs Western medicine

Figure S13 acupuncture vs Western medicine

Figure S14 bloodletting-cupping vs Western medicine

Figure S15 acupoint embedding vs Western medicine

Figure S16 electroacupuncture vs Western medicine

**Sensitivity analysis of adverse reactions figure S17～S19**

Figure S17 acupoint injection + Western medicine vs Western medicine

Figure S18 bloodletting-cupping vs Western medicine

Figure S19 acupuncture + Western medicine vs Western medicine
