# Supplementary material for: Comparison of Efficacy of Acupuncture-Related Therapy in the Treatment of Postherpetic Neuralgia: A Network Meta-Analysis of Randomized Controlled Trials
Source: Evid Based Complement Alternat Med. 2022 Oct 14;2022:3975389. doi: 10.1155/2022/3975389 (PMC9586726; doi:10.1155/2022/3975389)
Supplement: Supplementary Materials — Table S1: the PRISMA checklist. Table S2: Direct comparison of meta-analysis results. Figure S1∼S10: Sensitivity analysis of pain scores. Figure S11∼S16: Sensitivity analysis of total efficiency. Figure S17∼S19: Sensitivity analysis of adverse reactions. [file 3975389.f1.zip › Table S2. Direct comparison of meta analysis results.docx]

| **Table S2. Direct comparison of meta-analysis results** | | | | | | |  |  |  |  |  |  |
| --- | --- | --- | --- | --- | --- | --- | --- | --- | --- | --- | --- | --- |
| Outcome index | Comparison category | Number of studies | Heterogeneity | | Meta analysis results | |  |  |  |  |  |  |
|  |  |  | *I^2^* | *P* | MD/SMD, 95%CI | *P* |  |  |  |  |  |  |
| pain scores |  |  |  |  |  |  |  |  |  |  |  |  |
|  | electroacupuncture vs western medicine | 2 | 0% | 0.88 | **-1.57( -2.14, -0.99)** | **<0.00001** |  |  |  |  |  |  |
|  | warm acupuncture vs western medicine | 2 | 0% | 0.85 | **-1.71(-2.21, -1.29)** | **<0.00001** |  |  |  |  |  |  |
|  | bloodletting-cupping + western medicine vs western medicine | 3 | 14% | 0.31 | **-1.69(-1.96, -1.43)** | **<0.00001** |  |  |  |  |  |  |
|  | acupuncture + western medicine vs western medicine | 6 | 87% | <0.00001 | **-1.38(-1.98, -0.78)** | **<0.00001** |  |  |  |  |  |  |
|  | acupuncture vs western medicine | 3 | 90% | <0.00001 | -0.91(-2.14,0.32) | 0.15 |  |  |  |  |  |  |
|  | fire acupuncture vs western medicine | 2 | 0% | 0.89 | **-1.20( -1.62, -0.79)** | **<0.00001** |  |  |  |  |  |  |
|  | fire acupuncture + western medicine vs western medicine | 1 | NA | NA | -0.46(-1.14, -0.22) | 0.18 |  |  |  |  |  |  |
|  | bloodletting-cupping vs western medicine | 7 | 95% | <0.00001 | **-2.11(--3.10, -1.11)** | **<0.00001** |  |  |  |  |  |  |
|  | acupoint injection + western medicine vs western medicine | 2 | 99% | <0.00001 | -5.93(-15.68, 3.83) | 0.23 |  |  |  |  |  |  |
|  | acupoint embedding vs western medicine | 2 | 10% | 0.29 | **-0.52(-0.88, -0.15)** | **0.005** |  |  |  |  |  |  |
|  | acupoint injection vs western medicine | 1 | NA | NA | **-1.1(-1.53, -0.67)** | **<0.00001** |  |  |  |  |  |  |
|  | acupoint embedding + western medicine vs western medicine | 1 | NA | NA | **-0.66(-1.14, -0.18)** | **0.007** |  |  |  |  |  |  |
|  | electroacupuncture + western medicine vs western medicine | 2 | 80% | 0.03 | **-1.09(-1.87, -0.30)** | **0.007** |  |  |  |  |  |  |
|  | fire acupuncture vs acupuncture | 1 | NA | NA | 0.35(-0.38, -1.08) | 0.35 |  |  |  |  |  |  |
|  | fire acupuncture vs bloodletting-cupping | 1 | NA | NA | 0.05(-0.70, -0.80) | 0.90 |  |  |  |  |  |  |
|  | bloodletting-cupping vs acupuncture | 1 | NA | NA | -0.30(-1.07, 0.47) | 0.44 |  |  |  |  |  |  |
|  | acupuncture vs electroacupuncture | 1 | NA | NA | **-1.05(-2.00, -0.10)** | **0.03** |  |  |  |  |  |  |
|  | acupuncture + western medicine vs acupuncture | 1 | NA | NA | **-2.35(-3.18, -1.52)** | **<0.00001** |  |  |  |  |  |  |
| total efficiency |  |  |  |  |  |  |  |  |  |  |  |  |
|  | acupuncture + western medicine vs western medicine | 3 | 0% | 0.77 | **1.35(1.18, 1.53)** | **<0.00001** |  |  |  |  |  |  |
|  | fire acupuncture vs western medicine | 2 | 0% | 0.37 | **1.19(1.02, 1.39)** | **0.03** |  |  |  |  |  |  |
|  | acupuncture vs western medicine | 2 | 0% | 0.33 | **1.19 (1.01, 1.39)** | **0.04** |  |  |  |  |  |  |
|  | bloodletting-cupping vs western medicine | 4 | 78% | 0.003 | **1.11(1.05, 1.24)** | **0.001** |  |  |  |  |  |  |
|  | acupoint embedding vs western medicine | 2 | 0% | 0.86 | **1.19(1.02, 1.39)** | **0.03** |  |  |  |  |  |  |
|  | acupoint embedding + western medicine vs western medicine | 1 | NA | NA | 1.04(0.92, 1.18) | 0.52 |  |  |  |  |  |  |
|  | electroacupuncture vs western medicine | 2 | 87% | 0.006 | 1.15(0.83, 1.60) | 0.39 |  |  |  |  |  |  |
| adverse reactions | fire acupuncture vs acupuncture | 1 | NA | NA | 1.00(0.91, 1.10) | 1.00 |  |  |  |  |  |  |
|  | fire acupuncture vs bloodletting-cupping | 1 | NA | NA | 1.05(0.92, 1.20) | 0.47 |  |  |  |  |  |  |
|  | bloodletting-cupping vs acupuncture | 1 | NA | NA | 1.05(0.92, 1.20) | 0.47 |  |  |  |  |  |  |
|  | electroacupuncture vs acupuncture | 1 | NA | NA | 1.12(0.91, 1.38) | 0.30 |  |  |  |  |  |  |
|  |  |  |  |  |  |  |  |  |  |  |  |  |
|  | electroacupuncture vs western medicine | 1 | NA | NA | 0.11(0.01, 1.98) | 0.13 |  |  |  |  |  |  |
|  | warm acupuncture vs western medicine | 1 | NA | NA | 0.80 (0.28, 2.28) | 0.67 |  |  |  |  |  |  |
|  | bloodletting-cupping + western medicine vs western medicine | 1 | NA | NA | 0.92(0.40, 2.09) | 0.83 |  |  |  |  |  |  |
|  | acupoint injection + western medicine vs western medicine | 2 | 50% | 0.16 | 0.37(0.09, 1.56) | 0.18 |  |  |  |  |  |  |
|  | acupoint injection vs western medicine | 1 | NA | NA | 0.19 (0.01,3.82) | 0.28 |  |  |  |  |  |  |
|  | bloodletting-cupping vs western medicine | 2 | 0% | 0.60 | 1.10(0.85, 1.41) | 0.48 |  |  |  |  |  |  |
|  | electroacupuncture + western medicine vs western medicine | 1 | NA | NA | 1.05(0.35, 3.16) | 0.93 |  |  |  |  |  |  |
|  | acupuncture vs western medicine | 1 | NA | NA | 0.92(0.70, 1.21) | 0.57 |  |  |  |  |  |  |
|  | acupuncture + western medicine vs western medicine | 3 | 0% | 0.90 | 1.17 (0.42, 3.25) | 0.77 |  |  |  |  |  |  |
|  | acupoint embedding vs western medicine | 1 | NA | NA | 0.60(0.36, 1.00) | 0.05 |  |  |  |  |  |  |
| Abbreviation：NA, Data unavailable; MD, Mean Difference; SMD, Standard Mean Difference; CI, Confidence interval. The bold font indicates that there was a statistically significant difference between the two treatments. | | | | | | |  |  | 56% | 0.08 | **-0.86(-1.20, -0.52)** | **<0.00001** |
